# Contrasting losses and gains increases the predictability of behavior by frontal EEG asymmetry

**DOI:** 10.3389/fnbeh.2014.00149

**Published:** 2014-04-30

**Authors:** Ariel Telpaz, Eldad Yechiam

**Affiliations:** Max Wertheimer Minerva Center for Cognitive Studies, Faculty of Industrial Engineering and Management, Technion – Israel Institute of TechnologyHaifa, Israel

**Keywords:** frontal asymmetry, EEG, risk taking, losses, P300, fERN

## Abstract

Frontal asymmetry measured at rest using EEG is considered a stable marker of approach-avoidance behaviors and risk taking. We examined whether without salient cues of attention in the form of losses, predictability is reduced. Fifty-seven participants performed an experiential decision task in a gain-only, loss-only, and mixed (gains and losses) condition. Increased risk taking on the part of individuals with relatively high left frontal activation, as denoted by the Alpha band, was only observed in the task involving both gains and losses. Event-related potential analysis sheds light on the processes leading to this pattern. Left-frontal dominant individuals had increased fronto-central P300 activation following risky compared to safe outcomes, while right-frontal dominant individuals did not show a P300 difference following safe and risky outcomes. This interaction also only emerged when losses were contrasted with gains. The findings highlight the sensitivity of behavioral predictability to cues of valence.

## Introduction

One of the most prominent lines of research in predicting behavior from “at rest” physiological measures is the study of frontal EEG asymmetry. Davidson (1992, 1995) theorized that while both the left and right frontal regions are involved in the processing of emotions, left frontal regions are more active during approach-related emotions, whereas right frontal regions are more active during withdrawal related emotions. This hypothesis was confirmed by the finding that left frontal activity at rest is correlated with increased motivation to approach potentially positive stimuli (e.g., Davidson, 1992, 1995, 2004; Harmon-Jones and Allen, 1997; Sutton and Davidson, 1997, 2000; Coan et al., 2001; Coan and Allen, 2003, 2004)^1^ and increased risk taking (Knoch et al., 2006; Gianotti et al., 2009; Studer et al., 2013). More recently it was found that frontal EEG asymmetry is only modestly heritable (Bismark et al., 2010; Lee et al., 2011) and that it is highly sensitive to external stimuli such as music (Mikutta et al., 2012). However, no previous study has examined boundary conditions for the behaviors that could be predicted by frontal asymmetry. We take the approach that a substantial part of the variance of inter-individual differences in behavior is due to random influences, and examine whether salient cues of attention in the form of losses can increase the predictability of behavior.

A classical experiment by Kahneman and Tversky (1974) demonstrates the dependence of behavior on random cues. Participants were asked to indicate the percent of United Nations countries that were African national. Before they answered, they observed a roulette that was rigged to stop on either 10 or 65. Participants who observed the number 10 reported 25% on average, while those who saw the number 65 reported 45%. Hence, the cues in the environment which the subjects were randomly allocated to, affected much of their behavioral response. In a recent line of studies Ariely et al. (Ariely et al., 2003; Ariely and Norton, 2008) examined whether such random influences would affect real-world decisions, such as pricing of products. They found that presenting the last two digits of participants' social security number as an initial purchase offer had a huge effect on their valuation of a product. Ariely et al. (2003) further suggested that although much of our behavior is determined by random influences, people seek to coherently apply it to different conditions.

We examined whether the association between frontal EEG asymmetry and risk taking behavior would be more prominent with losses, a salient cue known to increase attention and decrease random responding (Taylor, 1991; Satterthwaite et al., 2007; Yechiam and Hochman, 2013). For example, previous studies have found that across different sessions, participants exhibited higher test-retest reliability in risk-taking tasks with losses than in tasks involving gains (Vlaev et al., 2009; Weller et al., 2011; Yechiam and Telpaz, 2013). Losses were also found to increase the association between low tonic arousal, which is considered a determinant of precarious behavior (Ellis, 1987; Zuckerman, 1994), and actual risk taking level (Yechiam and Telpaz, 2011). Therefore, we expected that the relationship between frontal asymmetry and risk taking would be stronger in tasks involving losses.

Following previous studies, (e.g., Harmon-Jones and Allen, 1997; Matsuda et al., 2013), we used the alpha band (8–13 Hz) to examine asymmetries in the frontal electrodes F3 and F4. In studies measuring simultaneous EEG and fMRI, frontal alpha power recorded during rest in these electrodes was found to be inversely related to the BOLD response of the corresponding region of the frontal cortex (Goldman et al., 2000; Laufs et al., 2003; Ritter and Villringer, 2006)^2^.

We further examined whether the presentation of losses impacts not only the behavioral responses to risk but also the cortical responses contingent upon being presented with risky outcomes. As with the behavioral results, it was assumed that the association between tonic arousal and acute cortical responses to risk would increase in task conditions involving losses because of the lower sensitivity to random noise in these conditions. First, we focused on the association between frontal asymmetry and the P300 event related potential (ERP). The P300 component it commonly thought to reflect cognitive operations related to attention and resource allocation (Donchin and Coles, 1988). Importantly, the P300 was found to be elevated following large compared to small outcomes (Yeung and Sanfey, 2004), possibly due to the increased significance of large outcomes (Gray et al., 2004). Moreover, individual differences in this tendency were found, with stronger P300 amplitudes for individuals with risk taking tendencies (Jia et al., 2013). To the extent that left frontal asymmetry is associated with risk taking, it was expected that left-dominant individuals would show larger P300 disparity between risky and safe outcomes, due to their increased exhilaration following large compared to small payoffs. Yet similarly to the relation between at-rest frontal asymmetry and behavior, we expected that this difference would be more prominent in task conditions involving losses.

Additionally, we also examined another ERP component directly associated with negative outcomes, the feedback-based Error Related negativity (fERN). This is a rapid fronto-central deflection which appears approximately 250 ms after experiencing losses, compared to equivalent gains (Gehring and Willoughby, 2002; Massar et al., 2012). Because approach motivation is associated with lower sensitivity to losses (Nash et al., 2012), we expected the fERN to be stronger for individuals with greater right frontal activity at rest.

## Materials and methods

### Participants

Fifty seven undergraduate students (30 males and 27 females) from the Technion, Israel institute of technology, participated in the study. Their mean age was 23.4 (*SD* = 2.3), ranging from 19 to 27. All of them were right handed, healthy, and free of neurological or psychiatric disorders. Participants were given a fixed fee of 90 New Israeli Shekel (NIS). In addition to their basic fee, they were also compensated according to the amount earned in the decision tasks. If a participant lost money, then the amount was deducted from his/her basic fee. The behavioral data of a larger subject population including the current subjects was reported in Yechiam and Telpaz (2013).

### Behavioral testing

We manipulated the valence of the outcomes by administering three repeated binary choice tasks, with either gains, losses, or mixed gains and losses. The All-Gains task was converted to an All-Losses task simply by multiplying all payoffs by -1. Because performing an All-Losses task may be discouraging (Yechiam and Hochman, 2013), we also added a Mixed task where a constant amount was reduced from each payoff of the All-Gains task, making some of the payoffs negative.

The participants performed all three tasks, with 60 trials per task. On every trial they had to select, using the mouse, one of two virtual buttons representing a Safe (S) and Risky (R) alternatives. The payoff distributions for each alternative in the three tasks were as follows:

**Table:** 

1. Mixed Task	
S	−5, 0, or 5 with equal probability
R	−25, −20, −15, 15, 20, or 25 with equal probability
2. All-Gains Task	
S	25, 30, or 35 with equal probability
R	5, 10, 15, 45, 50, or 55 with equal probability
3. All-Losses Task	
S	−35, −30, or −25 with equal probability
R	−55, −50, −45, −15, −10, −5 with equal probability

Alternatives S and R had equal expected values (in each task), but the variance of the outcome distribution was larger for R. In the Mixed task the outcomes involved both gains and losses. In the All-Gains (All-Losses) task a constant of 30 was added to (subtracted from) all payoffs so that the outcomes did not include any losses (gains). In each trial the choice outcomes were randomly sampled. The study used a within-subject design and the participants performed all three tasks in random order.

**Figure 1:**
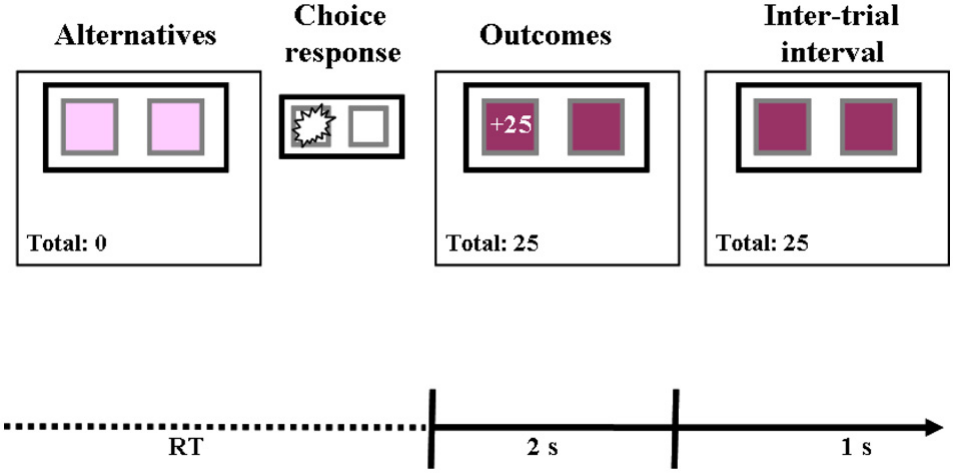
**An illustration of stimulus events in a single trial**. At the beginning of each trial two buttons were presented on the screen (Alternatives). The participants had to choose one of the buttons. Each selection was followed by a presentation of the obtained payoff on the selected alternative and the obtained-payoff counter for 2 s. After a 1 s interval the next trial began. Following each choice response the total payoff counter was updated. In this example the participant selected the left button and gained 25 points.

Prior to the beginning of the task, participants were informed that their assignment would be to repeatedly select between two buttons, and that some of their choices might be followed by gains and others by losses. They were also informed that they would perform three tasks and that their final take home amount would be determined by the accumulating score in one randomly determined task. However, they were not given any prior information as to the possible outcomes of selecting each button. As illustrated in Figure 1, following button selection the obtained payoff was presented on the selected button and on the obtained payoff counter. The button remained “selected” with the outcomes presented for 2 s. After a 1 s interval the next trial began. The right and left buttons were randomly assigned to the S and R alternative for each participant, thus controlling for the effect of position. This ordering was kept constant throughout the task.

Upon the beginning of the second and third tasks a message appeared with the information that the participant would now be performing a different task. At the end of the third task, participants were compensated according to the accumulated number of points earned in one randomly selected task, with a conversion rate of NIS 1 per 100 points earned. The main dependent measure was the proportion of risky selections across trials.

Additionally, we studied the effect of losses on participants' mean response time (RT). In decision tasks with no time requirements, response time is considered a resource (Bettman et al., 1990) which serves as an indication of elaborate processing (Horstmann et al., 2009; Hochman et al., 2010). We predicted that, as in previous studies, losses would result in increased RT (Porcelli and Delgado, 2009; Xue et al., 2009; Yechiam and Telpaz, 2011), thus potentially demarcating an increase in the attentional resource pool (Yechiam and Hochman, 2013).

### Data acquisition

Upon their arrival, participants were given a description of the study procedure and signed a consent form. We next recorded their EEG at rest. They were seated in a comfortable chair in a dimly lit soundproof room, and the experimenter communicated with them from an adjacent control room. During the recording participants were asked to relax and to minimize head and body movements. Two 2-min periods of resting EEG were recorded, one with eyes closed and one with eyes open. There was an interval of 30 s between recording periods. Five minutes after the end of the resting EEG measurement, participants began performing the decision tasks.

EEG was recorded using the ActiveTwo Biosemi system from 19 electrode sites on the scalp (Fp1, Fp2, F7, F3, Fz, F4, F8, T3, C3, Cz, C4, T4, T5, P3, Pz, P4, T6, O1, O2), mounted on an elastic cap (BioSemi, Amsterdam, the Netherlands) and placed according to the international 10–20 system. The system includes two additional electrodes, a Common Mode Sense (CMS) active electrode and a Driven Right Leg (DRL) passive electrode serving as ground. Active electrodes integrate the first amplification stage directly with the Ag/AgCl sensor, significantly reducing the effects of noise. The output ***impedance*** of the ***active*** sensor is smaller than 1Ω. To monitor artifacts originating from eye movements and blinks, four additional electrodes were used (two placed at lateral canthi and two below the eyes). In addition, two other electrodes, placed behind the ears, were used for offline referencing. Continuous EEG was recorded from 0.5–80 Hz and digitized at 256 Hz, using BioSemi ActiveView acquisition software.

### Data analysis

To compute the asymmetry index for each participant, the continuous EEG was divided into 2 s epochs (overlapping by 1.5 s). The data was visually inspected for artifacts, and epochs containing greater activity than 50 mV were rejected (on average 8% of the epochs were rejected). To derive power spectrum values the data was submitted to Fast Fourier Transform (FFT). Power was extracted in 0.5 Hz bins, which were combined into spectral bands. As noted above, for consistency with previous research (e.g., Lee et al., 2011; Matsuda et al., 2013), we focused on the alpha band (8–13 Hz) in the frontal electrodes F3 and F4^3^.

Because the distribution of power values tends to be positively skewed, alpha power values were natural log transformed (Ln) to normalize the data distribution. An index of hemispheric asymmetry was obtained by subtracting LnF4 from LnF3 values (LnF3–LnF4). As the within-subject correlation between the eyes open and eye closed measurement was very high (*r* = 0.82), we combined both measures into one frontal asymmetry index by averaging them (see e.g., Tomarken et al., 1990; Harmon-Jones and Allen, 1997). ERP analysis for the electrophysiological data recorded during the decision tasks was performed with EEGLAB 10.2.2 (Delorme and Makeig, 2004). The EEG data was filtered offline with a pass band from 0.5 to 40 Hz. Epochs were extracted for a time window of 800 ms post-stimulus, relative to a 200 ms pre-stimulus baseline. Artifacts caused by eye movements and muscular activity were removed using independent component analysis (ICA). Due to a technical problem there was no ERP data for one of the participants. We conducted a median split for the remaining 56 participants according to their frontal asymmetry score as measured prior to the decision tasks. Participants with higher alpha power at electrode site F3 (the left frontal electrode) were regarded as right frontal dominant while participants with higher alpha activation at electrode site F4 (the right frontal electrode) were considered as left frontal dominant. The median was very close to zero (−0.004 μV). Hence, one group involved 27 individuals with right frontal dominance and one individual with an asymmetry score practically at zero; and the other involved 28 individuals with left frontal dominance. We refer to the two groups as the right frontal dominant (RFD) group, and the left frontal dominant (LFD) group. In addition, we examined the LnF3–LnF4 score as a continuous predictor of individual differences.

To test for statistical differences in risk taking level between the two groups, we originally aimed to conduct an ANOVA with group (LFD vs. RFD) as a between-subjects factor and decision task as a within-subjects factor; yet due to differences in between-subject variance (see below) each decision task was separately analyzed. In addition, we examined the correlation between frontal asymmetry and risk taking in each decision task.

Our main ERP analysis compared the response to outcomes from the safe and risky alternatives as well as to relative gains and losses, and the effect of frontal asymmetry on these responses. In the first analysis, we focused on the time-window of 350–400 ms after the outcomes presentation. This time-window centered around the visually identified P300 component peak in the grand average waveforms. For analyzing the component peak, the maximum voltage within the corresponding time window was computed for each participant, separately for outcomes from the risky and safe options. To statistically test for differences in the P300 component, we used a mixed Analysis of Covariance (ANCOVA) with frontal asymmetry as a continuous between-subject factor and decision task and choice type (Safe vs. Risky) as within subject factors.

Additionally, for each of the three tasks we also examined the fERN component, centered around the visually identified peak 250–300 ms post stimulus, by comparing the ERP responses following relative losses (outcomes below the average of all outcomes) and relative gains (outcomes above the average of all outcomes). In this analysis as well the minimum voltage within the relevant time window was computed for each participant. A mixed ANCOVA was conducted as for the P300 analysis, with outcome valence replacing choice type as a within subject manipulation. In both ERP analyses we focused on the activity at the frontal electrode site Fz.

To estimate the neural generators of the brain activity associated with the difference between risky and safe choices, we used the standardized low-resolution electromagnetic tomography (sLORETA). This is a linear source localization method which computes the standardized current density with close to zero localization error (see Pascual-Marqui, 2002 for technical details). The analysis was based on the difference between amplitudes for relevant stimuli (e.g., risky minus safe outcomes for P300) at the 19 electrodes used in the study during the relevant time windows following outcome presentation.

## Results

### Behavioral results

The average proportion of risky choices across all trials was 0.46 (*SD* = 0.16) in the Mixed task, 0.49 (*SD* = 0.22) in the All-Gains task, and 0.44 (*SD* = 0.16) in the All-Losses task. There was no significant difference between task conditions [*F*_(1, 56)_ = 1.02, *p* = 0.32]. The finding of no difference in risk taking level between task conditions implies that, on average, losses did not affect the behavioral decision to take or avoid risk in this binary decision task. Thus, the participants did not show loss aversion, as recorded previously in repeated decision tasks with feedback (see review in Yechiam and Hochman, 2013).

Interestingly, the between-subject variances of the groups were somewhat different, with greater variance in the All-Gains task compared to the Mixed task [Levene *F*_(1, 112)_ = 4.15, *p* = 0.04] and All-Losses task [Levene *F*_(1, 112)_ = 6.73, *p* = 0.01]. No such differences were found between frontal asymmetry groups. Thus, the difference between tasks did not conform to the homoscedasticity assumption of the ANOVA, and in subsequent analyses involving different tasks we analyzed each task separately (by using *t*-tests).

An examination of response times (RT) showed that the average RT was 0.70 s (*SD* = 0.29) in the Mixed task, 0.61 s (*SD* = 0.17) in All-Gains task, and 0.65 s (*SD* = 0.21) in the All-Losses task. The difference in RT between the three tasks was significant [*F*_(2, 54)_ = 3.83, *p* = 0.03]. Furthermore, paired-sample *t*-tests showed that RT in the Mixed task was significantly longer than in the All-Gains task [*t*_(55)_ = 2.02, *p* = 0.048], but not significantly longer than in the All-Losses task [*t*_(55)_ = −0.93, *p* = 0.35]. The difference between the All-Losses and All-Gains tasks was not significant [*t*_(55)_ = 1.70, *p* = 0.1].

### Frontal asymmetry and risk taking

**Figure 2:**
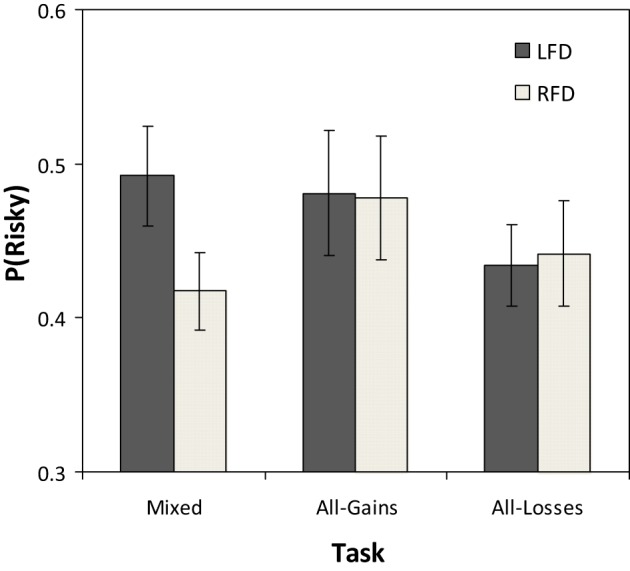
**Mean proportion of selections from the risky option across trials for the left frontal dominant (LFD) and right frontal dominant (RFD) groups in each of the three decision tasks**. The error bars represent the standard error of the mean.

A comparison of the average proportion of trials in which participants in the LFD and RFD groups chose the risky option in each task condition appears in Figure 2. In the Mixed task the average rate of risky selections in the LFD group was 0.50 (*SD* = 0.17) while in the RFD group it was 0.41 (*SD* = 0.13). The difference between groups in this task was significant [*t*_(54)_ = 2.19, *p* = 0.03]. In the All-Gains task, risk taking levels were similar in the two groups (LFD: *P*(R) = 0.48, *SD* = 0.21; RFD: *P*(R) = 0.47, *SD* = 0.21), and the difference between groups was not significant [*t*_(54)_ = 0.17, *p* = 0.86]. In the All-Losses task, as well, there were no significant differences between groups [LFD: *P*(R) = 0.43, *SD* = 0.14; RFD: *P*(R) = 0.44, *SD* = 0.18; *t*_(54)_ = −0.11, *p* = 0.90].

Additionally, we conducted correlational analyses that take into consideration individual variance within each group. The Pearson correlation between the pre-task frontal asymmetry and the rate of risky selections in the Mixed task was significant and negative (*r* = −0.30, *p* = 0.02), implying that participants with higher activation in the RFD tended to avoid risk. In the All-Gains task the correlation was weak and not significant (*r* = −0.11, *p* = 0.42), and in the All-Losses task the correlation was also not significant (*r* = −0.19, *p* = 0.16). These results were replicated using Spearman rank correlations; and also when controlling for the order of the respective task using partial correlations (Mixed: *r* = 0.30, *p* = 0.03; All-Gains: *r* = −0.10, *p* = 0.46; All losses: *r* = −0.18, *p* = 0.19).

### Frontal asymmetry and event related potentials

**Figure 3:**
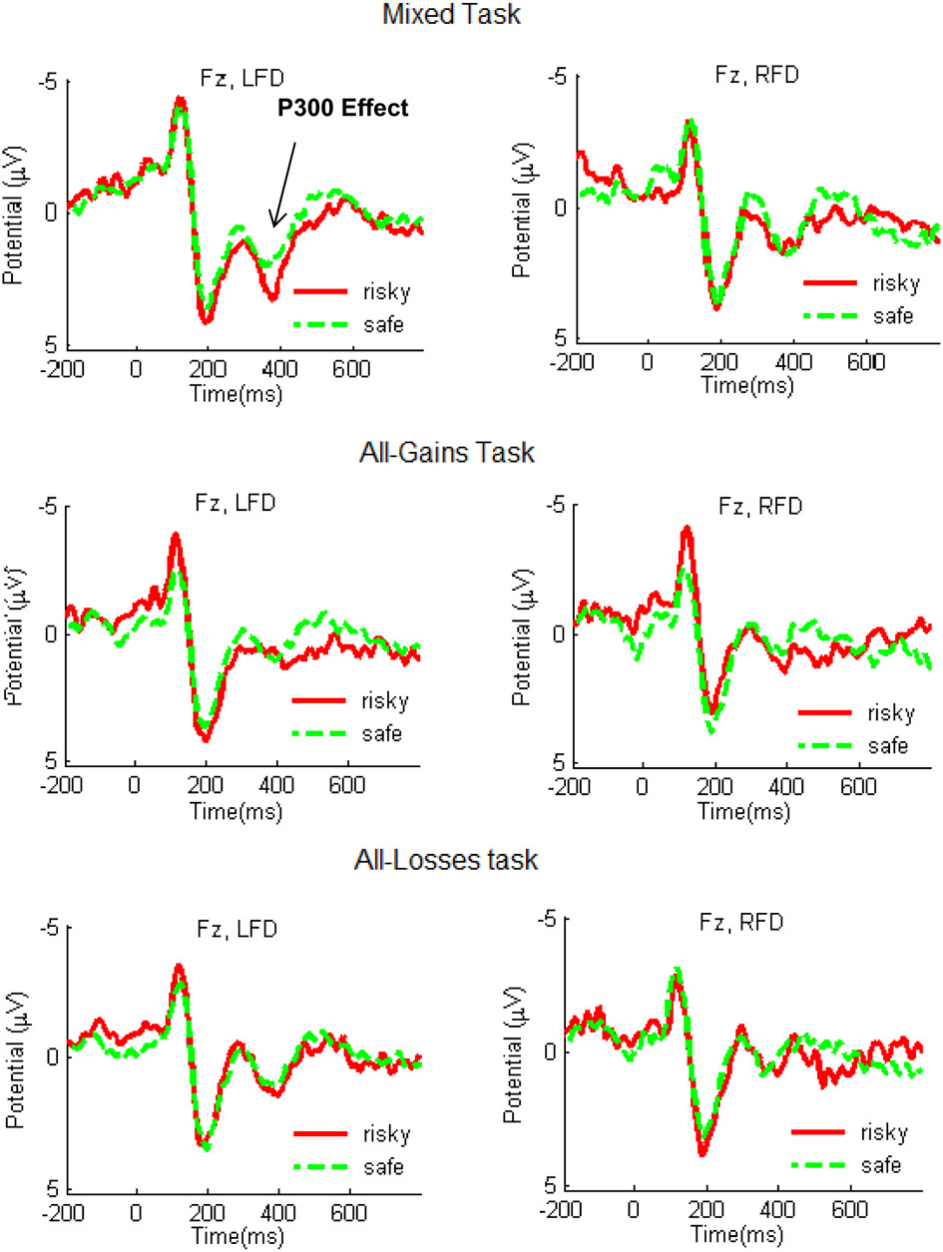
**Grand-average ERP waveforms recorded at electrode site Fz during the Mixed, All-Gains, and All-Losses tasks for the RFD (right column) and LFD groups**. The red line shows the average ERP response following outcomes from risky choices and the green dashed line indicates the response following outcomes from safe choices. A stronger P300 response following risky compared to safe outcomes emerged for the LFD group in the Mixed task.

Figure 3 illustrates the waveforms following risky and safe outcomes in the LFD and RFD groups. In order to examine differences in peak P300 responses we conducted a mixed ANCOVA with decision task and choice type (Safe vs. Risky) as within-subject factors and frontal asymmetry as a continuous between-subject factor. This analysis yielded a significant decision task by choice type by frontal asymmetry interaction [*F*_(2, 108)_ = 5.44, *p* = 0.006]. This result was replicated when controlling for task order by including the position of the Mixed and Loss tasks as additional covariates [*F*_(2, 108)_ = 5.84, *p* =0.004]. We proceeded by separately analyzing each of the decision tasks.

For the Mixed task there was no main effect of frontal asymmetry [*F*_(1, 54)_ = 0.03, *p* = 0.88] and a marginally significant effect of choice type [*F*_(1, 54)_ = 3.50, *p* = 0.067]. However, the interaction between frontal asymmetry and choice type was significant [*F*_(1, 54)_ = 4.46, *p* = 0.04]. *Post-hoc t*-tests with Bonferroni corrections for the median-split groups showed that the P300 response was significantly stronger following risky than following safe outcomes in the LFD group [*t*_(27)_ = 2.80, *p* < 0.01], but not in the RFD group [*t*_(27)_ = −0.35, *p* = 0.73]. Thus, in the Mixed task, only the LFD group showed increased P300 following outcomes from the risky alternative. None of the remaining interactions was significant.

The ANCOVA for the All-Gains task indicated no main effect of frontal asymmetry [*F*_(1, 54)_ = 0.92, *p* = 0.34] or choice type [*F*_(1, 54)_ = 0.70, *p* = 0.41], and no interaction between these two factors [*F*_(1, 54)_ = 2.62, *p* = 0.11]. In the All-Losses task there was also no main effect of frontal asymmetry [*F*_(1, 54)_ = 0.92, *p* = 0.34] or choice type [*F*_(1, 54)_ = 0.70, *p* = 0.41], and no interaction [*F*_(1, 54)_ = 2.61, *p* = 0.11]. Hence, the relation between frontal asymmetry predisposition and the P300 response only emerged in the mixed gain-loss setting.

**Figure 4:**
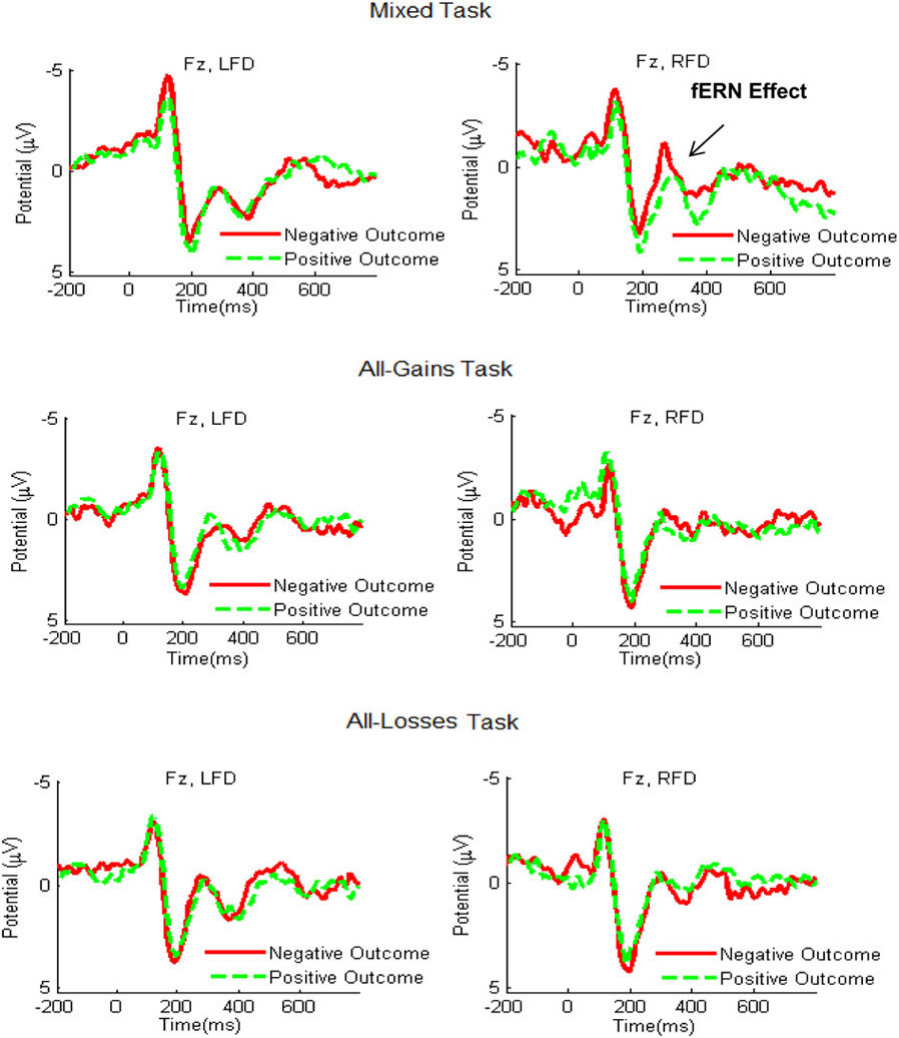
**Grand-average ERP waveforms recorded at electrode site Fz during the Mixed, All-Gains and All-Losses tasks for the RFD (right column) and LFD groups**. The red line shows the average ERP response in trials with relative losses and the green dashed line denotes trials with relative gains. The fERN effect, marking increased activation to losses compared to gains, was most distinct for the RFD group in the Mixed task.

We next examined the frontocentral responses to relative gains and relative losses (see Figure 4). A mixed ANCOVA for the fERN time window yielded no significant decision task by outcome valence by frontal asymmetry interaction [*F*_(2, 53)_ = 1.46, *p* = 0.24]. Moreover, a separate analyses of the Mixed tasks showed that the interaction of decision task by outcome valence was only marginally significant [*F*_(1, 54)_ = 3.00, *p* = 0.09]. This result was replicated when controlling for the order of the mixed task [*F*_(1, 53)_ = 2.90, *p* = 0.09]. We thus did not proceed further in conducting *post-hoc* tests for the different frontal asymmetry groups. For control purposes, we also analyzed the P300 results by outcome valence (following relative gains vs. losses) and the fERN results by choice type (following safe or risky outcomes). Both analyses showed no significant results.

**Figure 5:**
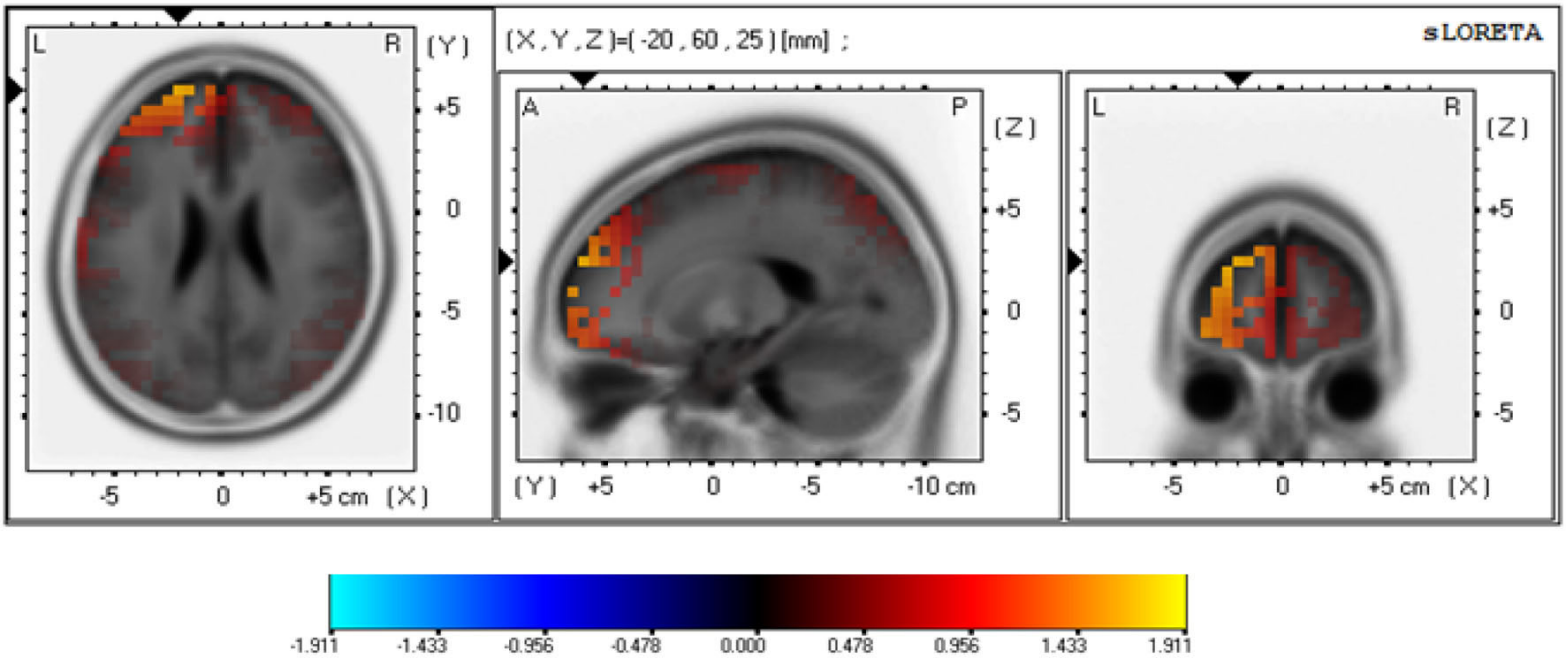
**sLORETA-based maps illustrating the density differences between the two frontal asymmetry groups at the P300 peak amplitudes**. The Talairach slices illustrate the estimated source of localized maximal activity of the P300_Risky-Safe_ effect in the Mixed task for the LFD group. sLORETA localized the source of activity in BA 10 of the middle frontal gyrus. Warm colors indicate higher current density (A/m^2^.10^−2^) following risky than safe outcomes.

Source localization analysis for the P300 (using sLORETA) focused on the LFD group's performance in the Mixed task, where a significant difference was observed following safe and risky outcomes. As shown in Figure 5, the estimated maximal activity of for the time window of the P300 response (350–400 ms) in the LFD group was located at Brodmann area 10 of the middle frontal gyrus (*x* = −20, *y* = 60, *z* = 25). A paired *t*-test revealed that for LFD individuals, at this location the difference between the brain response following safe and risky choices was significant [*t*_(27)_ = 2.76, *p* = 0.01]^4^.

## Discussion

Over the last 15 years it has become clear that there is a link between frontal asymmetry and choice behavior. The pioneering studies in this area have shown that greater right frontal EEG activity at rest characterizes individuals with low behavioral activation (Davidson, 1992, 1995; Harmon-Jones and Allen, 1997; Sutton and Davidson, 1997; Coan and Allen, 2003), and, in some studies, also higher behavioral inhibition (e.g., Sutton and Davidson, 1997). This led to the discovery of a link between frontal asymmetry and risk taking (Knoch et al., 2006; Gianotti et al., 2009; Studer et al., 2013). Risky situations, which are indistinct in terms of their overall value but have higher variance and greater polarity of outcomes, seem to provide a behavioral testing ground for approach and avoidance tendencies (Goudriaan et al., 2006; Suhr and Tsanadis, 2007; Demaree et al., 2008). Still, the boundaries of the relation between frontal hemispheric asymmetry and risk taking have hardly been examined. In the current paper, we suggested that the predictive value of frontal asymmetry is reduced in risk taking tasks without salient cues of attention, in the form of losses.

Our results showed that the predicted relation between frontal asymmetry and risk taking was only significant in a risk taking task involving both gains and losses. This relation did not emerge in a task involving only gains. In previous studies (e.g., Gianotti et al., 2009; Studer et al., 2013) frontal asymmetry predicted risk taking in a gain-domain task. However, in both of these studies the task also included the consequence of zero (losing all gains), which may lead to an orienting effect similar to that of losses.

Interestingly, the association between frontal asymmetry and risk taking was also considerably lower (and not significant) in the condition where all outcomes were losses. This finding may be explained by the suggestion that a loss is more salient when introduced against a reference point of a gain (Loomes and Sugden, 1982; Kahneman and Miller, 1986). For instance, under regret theory such a presentation facilitates the regret associated with the loss (Loomes and Sugden, 1982), which may in turn increase its salience and its effects on behavioral predictability. Alternatively, the null finding in the all-loss condition can be explained by the suggestion that a choice environment involving all losses may result in learned helplessness (Yechiam and Hochman, 2013), thereby having negative effects on the investment of attention toward the task (Alloy and Abramson, 1979). Under both accounts gains and not only losses increase the relation between physiological predispositions, such as frontal EEG asymmetry, and overt behavioral responses.

An analysis of event related potentials provided further support for the moderating role of losses. Greater left frontal activation level at rest was associated with increased fronto-central activation following outcomes from the risky alternative (compared to the safe alternative) within the P300 component; but this relation was only observed in the Mixed task with gains and losses. sLORETA (Pascual-Marqui, 2002) located the source of this effect at the middle frontal gyrus (MFG), which is part of BA 10, an area in which activation patterns were previously found to be related to risk sensitivity (Rogers et al., 1999; Schonberg et al., 2012). In other studies Brodmann area 10 has been linked to processes related to exploratory behavior (Boorman et al., 2009; Badre et al., 2012), which shares some of the features of risk task behavior (e.g., the need to balance potential benefits and adverse consequences). Related research has suggested that the BA 10 has an important role in the integration of cues with opposing outcomes (see Burgess et al., 2007; Koechlin and Hyafil, 2007). Our results add to this by demonstrating that the activation pattern in this area is influenced by individual differences, with stronger P300 responses for individuals with left frontal dominance at rest. However, these individual differences are only triggered with losses. It should be noted, though, that our source localization analyses used only 19 electrodes, which might have contributed to a limited spatial resolution of the source estimations.

By contrast, right frontal asymmetry was not associated with greater fronto-central activation following negative outcomes at the fERN component. This is inconsistent with previous findings suggesting that the fERN amplitude is a marker for processes mediating the association between resting EEG activity and risk taking behavior (Massar et al., 2012). However, our findings did show marginally significant results in the direction of greater fERN effect for the right frontal dominance group. Thus, the differences might be due to the smaller number of trials in the present investigation.

To summarize, our results showed that a significant relation between a neural predisposition to take risk and actual risk taking only emerged in the condition involving both gains and losses, a condition in which participants also invested the most time in the task. Additionally, the ERP results suggest that left dominant individuals showed more exhilaration following risky outcomes, but only in the task involving both gains and losses. These findings are consistent with our main argument that losses increase the reliability of the link between frontal asymmetry and risk taking behavior. Yet they also suggest that a payoff regime including only losses may lead to a breakup in these relations.

The results seem to be captured by Davidson's (1998) model in which the medial prefrontal cortex plays an important role in maintaining representations of behavioral-reinforcement contingencies in working memory. The embedded asymmetries in approach and avoidance are maintained as long as there is a steady attentional stream leading to the maintenance of task representations in working memory. In the absence of orienting stimuli this stream is interrupted and correlations between the frontal predisposition and actual behavior are attenuated. Nevertheless, this limitation concerning the relation between frontal predispositions and behavioral expressions was not previously taken notice of. We believe that only by taking such boundary conditions into consideration can the research into tonic physiological factors make claims that are of general predictive value.

The current findings are consistent with our previous results showing that the link between tonic arousal and risk taking is increased in tasks conditions with losses (Yechiam and Telpaz, 2011). Furthermore, they may serve to explain the increased relation between genetic predisposition and risk taking in tasks with losses (e.g., Crisan et al., 2009; Zhong et al., 2009). For example, Crisan et al. (2009) focused on one of the presumed genetic markers of risk taking, the short version of the serotonin transporter (5-HTTLPR) polymorphism. Their results showed that carriers of the short allele (s-carriers) did not show a different risk taking pattern when the task included only gains, but they exhibited increased risk taking when one of the gains was framed as a loss. Our theoretical model and findings suggest that this interaction may be another case where individual differences are highly affected by random processes, but become more predictable when a task includes both gains and losses.

### Conflict of interest statement

The authors declare that the research was conducted in the absence of any commercial or financial relationships that could be construed as a potential conflict of interest.

## Footnotes

^1^In some studies right frontal activity was also found to be correlated with increased motivation to withdraw from negative stimuli (e.g., Davidson, 1998) but this was not replicated in other studies (e.g., Harmon-Jones and Allen, 1997; Coan and Allen, 2004).

^2^Although it is widely accepted in the literature on frontal asymmetry that baseline alpha power is inversely related to frontal cortical activation, it appears that alpha oscillations also play an active role in information processing tasks requiring the suppression of attention (for a review see Klimesch, 2012).

^3^We measured absolute alpha power (i.e., we did not divide it by the power of other frequency bands).

^4^This statistical test should be qualified, however, for the fact that the sLORETA tests for differences in a solution space that contains more than 6000 voxels.

## References

[B1] Alloy L. B. Abramson L. Y. (1979). Judgment of contingency in depressed and nondepressed students: sadder but wiser? J. Exp. Psychol. Gen. 108, 441–485 10.1037/0096-3445.108.4.441 528910

[B2] Ariely D. Loewenstein G. Prelec D. (2003). “Coherent arbitrariness”: stable demand curves without stable preferences. Q. J. Econ. 118, 73–106 10.1162/00335530360535153

[B3] Ariely D. Norton M. I. (2008). How actions create – not just reveal – preferences. Trends Cogn. Sci. 12, 13–16 10.1016/j.tics.2007.10.008 18063405

[B4] Badre D. Doll B. B. Long N. M. Frank M. J. (2012). Rostrolateral prefrontal cortex and individual differences in uncertainty-driven exploration. Neuron 73, 595–607 10.1016/j.neuron.2011.12.025 22325209 PMC3285405

[B5] Bettman J. R. Johnson E. J. Payne J. W. (1990). A componential analysis of cognitive effort in choice. Organ. Behav. Hum. Dec. 45, 111–139 10.1016/0749-5978(90)90007-V2364741

[B6] Bismark A. W. Moreno F. A. Stewart J. L. Towers D. N. Coan J. A. Oas J. (2010). Polymorphisms of the HTR1a allele are linked to frontal brain electrical asymmetry. Biol. Psychol. 83, 153–158 10.1016/j.biopsycho.2009.12.002 20025927 PMC2845287

[B7] Boorman E. D. Behrens T. E. Woolrich M. W. Rushworth M. F. (2009). How green is the grass on the other side? Frontopolar cortex and the evidence in favor of alternative courses of action. Neuron 62, 733–743 10.1016/j.neuron.2009.05.014 19524531

[B8] Burgess P. W. Dumontheil I. Gilbert S. J. (2007). The gateway hypothesis of rostral prefrontal cortex (area 10) function. Trends Cogn. Sci. 11, 290–298 10.1016/j.tics.2007.05.004 17548231

[B9] Coan J. A. Allen J. J. B. (2003). Frontal EEG asymmetry and the behavioral activation and inhibition systems. Psychophysiology 40, 106–114 10.1111/1469-8986.00011 12751808

[B10] Coan J. A. Allen J. J. B. (2004). Frontal EEG asymmetry as a moderator and mediator of emotion. Biol. Psychol. 67, 7–49 10.1016/j.biopsycho.2004.03.002 15130524

[B11] Coan J. A. Allen J. J. B. Harmon-Jones E. (2001). Voluntary facial expression and hemispheric asymmetry over the frontal cortex. Psychophysiology 38, 912–925 10.1111/1469-8986.3860912 12240668

[B12] Crisan L. G. Pana S. Vulturar R. Heilman R. M. Szekely R. Druga B. Dragos N. (2009). Genetic contributions of the serotonin transporter to social learning of fear and economic decision making. Soc. Cogn. Affect. Neurosci. 4, 399–408 10.1093/scan/nsp019 19535614 PMC2799947

[B13] Davidson R. J. (1992). Anterior cerebral asymmetry and the nature of emotion. Brain Cogn. 20, 125–151 10.1016/0278-2626(92)90065-T 1389117

[B14] Davidson R. J. (1995). Cerebral asymmetry, emotion, and affective style, in Brain Asymmetry, eds Davidson R. J. Hugdahl K. (Cambridge, MA: MIT Press), 361–387

[B15] Davidson R. J. (1998). Affective style and affective disorders: perspectives from affective neuroscience. Cogn. Emot. 12, 307–330 10.1080/026999398379628

[B16] Davidson R. J. (2004). What does the prefrontal cortex “do” in affect: perspectives on frontal EEG asymmetry research. Biol. Psychiatry 67, 219–223 10.1016/j.biopsycho.2004.03.008 15130532

[B17] Delorme A. Makeig S. (2004). EEGLAB: an open source toolbox for analysis of single-trial EEG dynamics. J. Neurosci. Methods 134, 9–21 10.1016/j.jneumeth.2003.10.009 15102499

[B18] Demaree H. A. DeDonno M. A. Burns K. J. Everhart D. E. (2008). You bet: how personality differences affect risk taking preferences. Pers. Indiv. Differ. 44, 1484–1494 10.1016/j.paid.2008.01.005

[B19] Donchin E. Coles M. G. H. (1988). Is the P300 component a manifestation of cognitive updating? Behav. Brain Sci. 11, 357–427 10.1017/S0140525X00058027

[B20] Ellis L. (1987). Relationships of criminality and psychopathy with eight other apparent behavioral manifestations of sub-optimal arousal. Pers. Indiv. Differ. 8, 905–925 10.1016/0191-8869(87)90142-5

[B22] Gehring W. J. Willoughby A. R. (2002). The medial frontal cortex and the rapid processing of monetary gains and losses. Science 295, 2279–2282 10.1126/science.1066893 11910116

[B23] Gianotti L. R. Knoch D. Faber P. L. Lehmann D. Pascual-Marqui R. D. Diezi C. Schoch C. (2009). Tonic activity level in the right prefrontal cortex predicts individuals' risk taking. Psychol. Sci. 20, 33–38 10.1111/j.1467-9280.2008.02260.x 19152538

[B24] Goldman R. I. Stern J. M. Engel J. Jr. Cohen M. S. (2000). Acquiring simultaneous EEG and functional MRI. Clin. Neurophysiol. 111, 1974–1980 10.1016/S1388-2457(00)00456-9 11068232

[B25] Goudriaan A. E. Oosterlaan J. de Beurs E. van den Brink W. (2006). Neurocognitive functions in pathological gambling: A comparison with alcohol dependence, Tourette syndrome and normal controls. Addiction 101, 534–547 10.1111/j.1360-0443.2006.01380.x 16548933

[B27] Gray H. M. Ambady N. Lowenthal W. T. Deldin P. (2004). P300 as an index of attention to self-relevant stimuli. J. Exp. Soc. Psychol. 40, 216–224 10.1016/S0022-1031(03)00092-1

[B28] Harmon-Jones E. Allen J. J. B. (1997). Behavioral activation sensitivity and resting frontal EEG asymmetry: covariation of putative indicators related to risk for mood disorders. J. Abnorm. Psychol. 106, 159–163 10.1037/0021-843X.106.1.159 9103728

[B29] Hochman G. Ayal S. Glöckner A. (2010). Physiological arousal in processing recognition information: Ignoring or integrating cognitive cues? Judg. Decis. Making 5, 285–299 Available online at: http://journal.sjdm.org/10/rh9/rh9.html

[B30] Horstmann N. Ahlgrimm A. Glöckner A. (2009). How distinct are intuition and deliberation? An eye-tracking analysis of instruction-induced decision modes. Judg. Decis. Making 4, 335–354 Available online at: http://journal.sjdm.org/9323/jdm9323.pdf

[B31] Jia S. Zhang W. Li P. Feng T. Li H. (2013). Attitude toward money modulates outcome processing: An ERP study. Soc. Neurosci. 8, 43–51 10.1080/17470919.2012.713316 22856426

[B32] Kahneman D. Miller D. T. (1986). Norm theory: Comparing reality to its alternatives. Psychol. Rev. 93, 136–153 10.1037/0033-295X.93.2.136

[B33] Kahneman D. Tversky A. (1974). Judgments under uncertainty: Heuristics and biases. Science 185, 1121–1131 10.1126/science.185.4157.1124 17835457

[B34] Klimesch W. (2012). Alpha-band oscillations, attention, and controlled access to stored information. Trends Cogn. Sci. 16, 606–617 10.1016/j.tics.2012.10.007 23141428 PMC3507158

[B35] Knoch D. Gianotti L. R. R. Pascual-Leone A. Treyer V. Regard M. Hohmann M. (2006). Disruption of right prefrontal cortex by low-frequency repetitive transcranial magnetic stimulation induces risk-taking behavior. J. Neurosci. 26, 6469–6472 10.1523/JNEUROSCI.0804-06.2006 16775134 PMC6674035

[B36] Koechlin E. Hyafil A. (2007). Anterior prefrontal function and the limits of human decision-making. Science 318, 594–598 10.1126/science.1142995 17962551

[B37] Laufs H. Kleinschmidt A. Beyerle A. Eger E. Salek-Haddadi A. Preibisch C. (2003). EEG-correlated fMRI of human alpha activity. Neuroimage 19, 1463–1476 10.1016/S1053-8119(03)00286-6 12948703

[B38] Lee T.-W. Yu Y. W. Y. Hong C.-J. Tsai S.-J. Wu H.-C. Chen T.-J. (2011). The influence of serotonin transporter polymorphisms on cortical activity: a resting EEG study. BMC Neurosci. 12:33 10.1186/1471-2202-12-33 21507249 PMC3110125

[B39] Loomes G. Sugden R. (1982). Regret theory: an alternative theory of rational choice under uncertainty. Econ. J. 92, 805–824 10.2307/2232669

[B40] Massar S. A. A. Rossi V. Schutter D. J. L. G. Kenemans J. L. (2012). Baseline EEG theta/beta ratio and punishment sensitivity as biomarkers for feedback-related negativity (FRN) and risk-taking. Clin. Neurophysiol. 123, 1958–1965 10.1016/j.clinph.2012.03.005 22542439

[B41] Matsuda I. Nittono H. Allen J. J. (2013). Detection of concealed information by P3 and frontal EEG asymmetry. Neurosci. Lett. 537, 55–59 10.1016/j.neulet.2013.01.029 23370285

[B42] Mikutta C. Altorfer A. Strik W. Koenig T. (2012). Emotions, arousal, and frontal alpha rhythm asymmetry during Beethoven's 5th symphony. Brain Topogr. 25, 423–430 10.1007/s10548-012-0227-0 22534936

[B43] Nash K. N. Inzlicht M. McGregor I. D. (2012). Approach-related left prefrontal EEG asymmetry predicts muted error-related negativity. Biol. Psychol. 81, 96–102 10.1016/j.biopsycho.2012.05.005 22634389

[B44] Pascual-Marqui R. D. (2002). Standardized low resolution brain electromagnetic tomography (sLORETA): technical details. Methods Find. Exp. Clin. 24, 5–12 12575463

[B46] Porcelli A. J. Delgado M. R. (2009). Acute stress modulates risk taking in financial decision making. Psychol. Sci. 20, 278–283 10.1111/j.1467-9280.2009.02288.x 19207694 PMC4882097

[B47] Ritter P. Villringer A. (2006). Simultaneous EEG–fMRI. Neurosci. Biobehav. Rev. 30, 823–838 10.1016/j.neubiorev.2006.06.008 16911826

[B48] Rogers R. O. Owen A. M. Middleton H. C. Williams E. J. Pickard J. D. Sahakian B. J. (1999). Choosing between small, likely rewards and large, unlikely rewards activates inferior and orbital prefrontal cortex. J. Neurosci. 19, 9029–9038 10516320 10.1523/JNEUROSCI.19-20-09029.1999 PMC6782753

[B49] Satterthwaite T. D. Green L. Myerson J. Parker J. Ramaratnam M. Buckner R. L. (2007). Dissociable but inter-related systems of cognitive control and reward during decision making: evidence from pupillometry and event-related fMRI. Neuroimage 37, 1017–1031 10.1016/j.neuroimage.2007.04.066 17632014

[B50] Schonberg T. Fox C. R. Mumford J. A. Congdon E. Trepel C. Poldrack R. A. (2012). Decreasing ventromedial prefrontal cortex activity during sequential risk-taking: an fMRI investigation of the balloon analogue risk task. Front. Neurosci. 6:80 10.3389/fnins.2012.00080 PMC3366349 22675289

[B51] Studer B. Pedroni A. Rieskamp J. (2013). Predicting risk-taking behavior from prefrontal resting-state activity and personality. PLoS ONE 8:e76861 10.1371/journal.pone.0076861 24116176 PMC3792091

[B52] Suhr J. A. Tsanadis J. (2007). Affect and personality correlates of the Iowa Gambling Task. Pers. Indiv. Differ. 43, 27–36 10.1016/j.paid.2006.11.004

[B53] Sutton S. K. Davidson R. J. (1997). Prefrontal brain asymmetry: a biological substrate of the behavioral approach and inhibition systems. Psychol. Sci. 8, 204–210 10.1111/j.1467-9280.1997.tb00413.x

[B54] Sutton S. K. Davidson R. J. (2000). Prefrontal brain electrical asymmetry predicts the evaluation of affective stimuli. Neuropsychologia 38, 1723–1733 10.1016/S0028-3932(00)00076-2 11099730

[B55] Taylor S. E. (1991). The asymmetrical impact of positive and negative events: the mobilization-minimization hypothesis. Psychol. Bull. 110, 67–85 10.1037/0033-2909.110.1.67 1891519

[B57] Tomarken A. J. Davidson R. J. Henriques J. B. (1990). Resting frontal brain asymmetry predicts affective responses to films. J. Pers. Soc. Psychol. 59, 791–801 10.1037/0022-3514.59.4.791 2254854

[B58] Vlaev I. Chater N. Stewart N. (2009). Dimensionality of risk perception: factors affecting consumer understanding and evaluation of financial risk. J. Behav. Finance 10, 158–181 10.1080/15427560903167720

[B59] Weller J. A. Levin I. P. Denburg N. (2011). Trajectory of risky decision making for potential gains and losses from ages 5 to 85. J. Behav. Decis. Making 24, 331–344 10.1002/bdm.690

[B60] Xue G. Lu Z. Levin I. P. Weller J. A. Li X. Bechara A. (2009). Functional dissociations of risk and reward processing in the medial prefrontal cortex. Cereb. Cortex 19, 1019–1027 10.1093/cercor/bhn147 18842669 PMC2665154

[B61] Yechiam E. Hochman G. (2013). Losses as modulators of attention: review and analysis of the unique effects of losses over gains. Psychol. Bull. 139, 497–518 10.1037/a0029383 22823738

[B62] Yechiam E. Telpaz A. (2011). To take risk is to face loss: a tonic pupillometry study. Front. Psychol. 2:344 10.3389/fpsyg.2011.00344 22125546 PMC3222224

[B63] Yechiam E. Telpaz A. (2013). Losses induce consistency in risk taking even without loss aversion. J. Behav. Decis. Making 26, 31–40 10.1002/bdm.758

[B64] Yeung N. Sanfey A. G. (2004). Independent coding of reward magnitude and valence in the human brain. J. Neurosci. 24, 6258–6264 10.1523/JNEUROSCI.4537-03.2004 15254080 PMC6729539

[B65] Zhong S. Israel S. Xue H. Sham P. C. Ebstein R. P. Chew S. H. (2009). A neurochemical approach to valuation sensitivity over gains and losses. Philos. Trans. R. Soc. Lond. B Biol. Sci. 276, 4181–4188 10.1098/rspb.2009.1312 19726478 PMC2821348

[B66] Zuckerman M. (1994). Behavioral Expressions and Biosocial Bases of Sensation Seeking. New York, NY: Cambridge University Press

